# A FEM-Based Machine Learning Framework for Online Stress Field Estimation in Hydropower Regulating Rings

**DOI:** 10.3390/s26165129

**Published:** 2026-08-13

**Authors:** Ronny Francis Ribeiro Junior, Paulo Henrique Favero Loss, Bruno Correia Macedo, Frederico de Oliveira Assuncao, Erik Leandro Bonaldi, Luiz Eduardo Borges-da-Silva

**Affiliations:** 1 R&D Department, Gnarus Institute, Itajuba 37500-052, MG, Brazil; 2 Sinop Energia, Sinop 78556-024, MT, Brazil; paulo.loss@sinopenergia.com.br (P.H.F.L.); bruno.macedo@sinopenergia.com.br (B.C.M.); 3 R&D Department, PS Soluções, Itajubá 37502-485, MG, Brazil; fred@pssolucoes.com.br (F.d.O.A.); erik@pssolucoes.com.br (E.L.B.); leborges@unifei.edu.br (L.E.B.-d.-S.)

**Keywords:** machine learning, finite element method, PCA, Random Forest, hydropower regulating ring, structural health monitoring

## Abstract

Data-driven surrogate methods are increasingly applied to real-time structural health monitoring of critical engineering systems, but their use is often limited by the computational cost of high-fidelity finite element (FEM) simulations. This work proposes an FEM-based machine learning framework combining FEM, Principal Component Analysis (PCA), and Random Forest regression to reconstruct full-field von Mises stress distributions of a hydropower regulating ring from a reduced set of proximity sensor measurements. A calibrated 3D FEM model generated a representative dataset of operating conditions using a Design of Experiments (DOE) sampling strategy, reducing the simulation space. The resulting stress fields were reduced using a single global PCA model, and the retained principal components were predicted by a single Random Forest model trained on the guide vane opening and four displacement sensors installed on the turbine unit. Stress reconstruction was obtained via inverse PCA transformation and validated against FEM results through a leave-one-opening-out cross-validation, in which each guide vane opening was entirely withheld from training. The method achieved an average PSNR of 17.4 dB and SSIM of 0.837 across the eleven withheld openings, with 8 to 9 PCA components sufficient to preserve over 95% of the cumulative explained variance. A sensitivity analysis of the ensemble size showed that 100 decision trees provide accuracy comparable to larger ensembles at lower computational cost, and a feature importance analysis revealed the guide vane opening as the dominant predictor, with the four sensors providing complementary, fine-grained corrections. The framework enables near real-time reconstruction, requiring approximately 1.5 s per condition versus several hours for FEM. These results show that combining physics-based modeling with machine learning enables efficient structural monitoring for predictive maintenance and operational decision-making in hydroelectric systems.

## 1. Introduction

Hydroelectric power generation plays a fundamental role in the global energy matrix, providing a renewable, dispatchable, and large-scale source of electricity that is essential for grid stability and the integration of variable renewable sources [1]. In Brazil, hydropower accounts for approximately 60% of the national installed capacity, making the reliable and efficient operation of hydroelectric generating units a matter of both economic and strategic importance [2]. In this context, equipment availability and operational efficiency are paramount concerns: unplanned outages not only interrupt generation but also incur substantial costs through lost revenue and regulatory penalties for unavailability, providing a persistent motivation for advances in condition monitoring and predictive maintenance.

Among the numerous mechanical components that compose a hydropower generating unit, the regulating ring, also referred to as the operating ring or distributor ring, plays a critical role in turbine control. Its function is to transmit the force exerted by the hydraulic servomotors simultaneously and equally to the levers and connecting rods of all guide vanes, thereby ensuring synchronous opening and closing of the distributor and precise regulation of turbine output [3]. By design, the ring operates under cyclic mechanical loading, as each start-up, shutdown, and load variation cycle imposes alternating forces and displacements on its structure. Over time, this repeated loading leads to progressive degradation mechanisms, including fatigue crack initiation, wear of self-lubricating bearing segments, plastic deformation, and loss of geometric alignment, all of which can ultimately compromise the operational integrity of the generating unit [4,5].

Monitoring the structural condition of the regulating ring is therefore of the utmost importance for early fault detection and the transition from reactive to prescriptive maintenance strategies. This can be accomplished through the installation of various sensor types, including proximity sensors to track radial and axial displacements, and hydraulic pressure transducers to characterize the loading state of the actuation cylinders. Such instrumentation allows for the continuous, synchronized acquisition of the ring’s mechanical response during operation, providing the data foundation for condition assessment [6,7,8].

However, the regulating ring is a large annular structure whose structural behavior is governed by a spatially distributed stress field that evolves across its entire circumference. Full coverage of this field through direct sensing would require a prohibitively large number of sensors, making comprehensive instrumentation both economically impractical and logistically challenging in an industrial plant environment. In practice, only a limited number of measurement points can be instrumented, leaving the majority of the component’s stress state unobserved [9].

One natural approach to bridge this gap is the use of the Finite Element Method (FEM), which enables the computation of the complete stress distribution throughout the ring given a set of boundary conditions, including the displacements and forces measured at sensor locations. A calibrated FEM model can serve as a physics-based surrogate, inferring the unobserved stress field from the available measurements [10]. This approach has been employed in structural health monitoring to complement sparse sensing with high-fidelity numerical predictions. However, a critical limitation of direct FEM-based inference is its computational cost: a single high-fidelity analysis of a detailed three-dimensional model can require several minutes to hours of computation time, making real-time or near-real-time execution during plant operation impractical [11].

Machine learning techniques offer a compelling solution to this bottleneck [12,13,14,15]. By training a data-driven surrogate model on a pre-computed library of FEM simulations, each associating a specific configuration of sensor readings with the corresponding full stress field, it becomes possible to replicate the predictive capability of the FEM model at a fraction of the inference cost. Once trained offline, the surrogate can evaluate new sensor inputs and produce stress field estimates in seconds, enabling genuine online monitoring [16,17]. This paradigm, combining a physics-based simulation engine with a data-driven inference layer, constitutes the foundation of an FEM-based machine learning framework that provides a continuously updated estimate of the physical asset’s current structural state and supports maintenance decision-making in real time [18,19,20]. Beyond predictive accuracy, however, the practical adoption of such surrogate models in an industrial setting also depends on their interpretability, that is, on the extent to which the contribution of each individual sensor to the reconstructed structural response can be understood and related to the physical behavior of the monitored component.

This paper proposes and implements an FEM-based machine learning framework for the regulating ring of a hydropower generating unit. A FEM model of the ring is used to generate a comprehensive simulation dataset covering a wide range of operational loading conditions. Principal Component Analysis is applied to reduce the dimensionality of the simulated stress fields, and a Random Forest (RF) regressor is trained to map displacement measurements from a small set of proximeters to the corresponding PCA scores, enabling reconstruction of the full von Mises equivalent stress distribution. In addition to reporting the predictive accuracy of the surrogate model, this work also examines the individual contribution of the guide vane opening and of each proximity sensor to the reconstructed stress field, combining the Random Forest feature importance with a complementary, model-agnostic sensor perturbation analysis, so as to provide a more interpretable and physically grounded assessment of the surrogate model’s behavior. The framework is demonstrated on Generating Unit 02 at the Sinop Energia (Sinop Hydroelectric Power Plant), Brazil, which is integrated into the plant’s SCADA-based supervisory system for continuous online visualization.

The remainder of this paper is organized as follows. Section 2 presents the materials and methods employed in the development of the proposed framework. Section 3 reports the experimental results, including the reconstruction accuracy, the sensor and opening contribution analyses, and the practical implications of the proposed methodology. Finally, Section 4 concludes the paper and highlights opportunities for future work.

## 2. Materials and Methods

This section describes the methodology adopted for the development of the proposed framework for the structural monitoring of the regulating ring. The methodology integrates field measurements, finite element modeling, and machine learning techniques to estimate the full-field stress distribution from a limited number of displacement measurements during plant operation.

The proposed workflow comprises six main stages. First, the hydroelectric generating unit and the regulating ring investigated in this study are presented. Subsequently, a detailed three-dimensional model of the regulating mechanism is developed based on engineering drawings, technical documentation, and field measurements. A planimetric and altimetric survey is then conducted to characterize the actual geometric condition of the regulating ring and provide reference measurements for both the numerical model and the installed proximity sensors. Based on the developed geometry and the measured structural condition, a finite element model is constructed to evaluate the stress distribution under different operating conditions. The numerical model is subsequently employed to define the sensor locations and to generate a representative simulation database using a Design of Experiments methodology. Finally, the generated database is used to train a machine learning surrogate model capable of reconstructing the complete stress field of the regulating ring in near real time from the proximity sensor measurements.

### 2.1. Case Study Description

The proposed framework was developed using the Generating Unit 02 of the Sinop Energia, located on the Teles Pires River in the northern region of Mato Grosso State, Brazil. The plant began commercial operation in 2019 and represents one of the most important hydroelectric generation assets in the region, contributing significantly to the Brazilian interconnected power system. The reservoir covers approximately 342 km^2^, spanning portions of the municipalities of Cláudia, Itaúba, Ipiranga do Norte, Sinop, and Sorriso. The plant has an installed capacity of 401.88 MW and is equipped with two Kaplan turbine-generator units operating under a reference net head of 27.6 m [21].

Kaplan turbines are widely employed in low-head, high-flow hydropower applications due to their adjustable runner blades and guide vanes, which provide high efficiency over a broad operating range. In such turbines, the regulation of water flow is achieved through a distributor mechanism composed of multiple guide vanes simultaneously actuated by hydraulic servomotors through a regulating ring. This component is responsible for transmitting the actuation forces uniformly to all guide vanes, ensuring synchronized opening and closing during startup, shutdown, and load-following operations.

**Figure 1:**
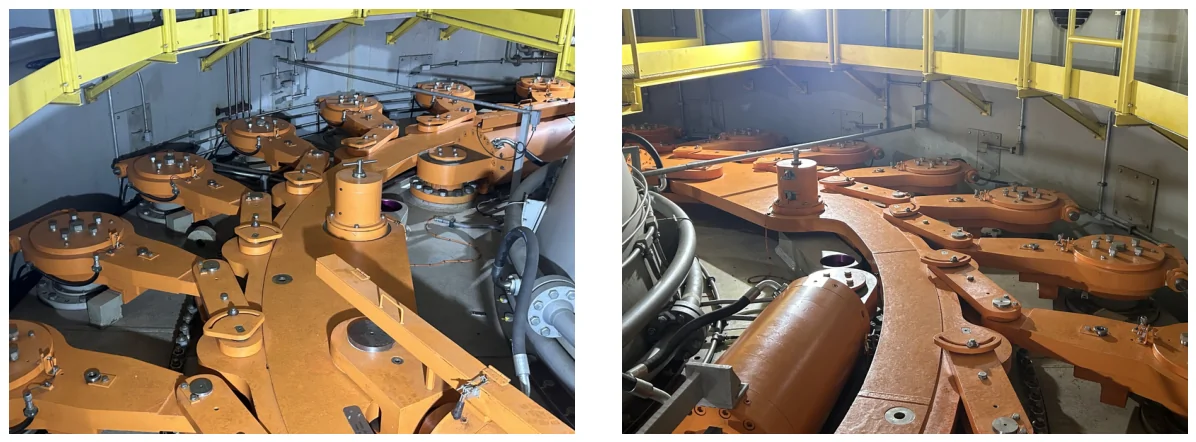
Regulating ring of Generating Unit 02 at the Sinop Energia.

The regulating ring investigated in this study belongs to Generating Unit 02. A photograph of the component installed in the turbine distributor mechanism is presented in Figure 1. Due to the critical role of the regulating ring in turbine operation, failures or excessive deformation may compromise guide vane synchronization, increase mechanical stresses, and ultimately affect turbine availability and reliability. Consequently, the component was selected as the target asset for the implementation of the proposed methodology.

### 2.2. Geometric Modeling of the Regulating Ring

**Figure 2:**
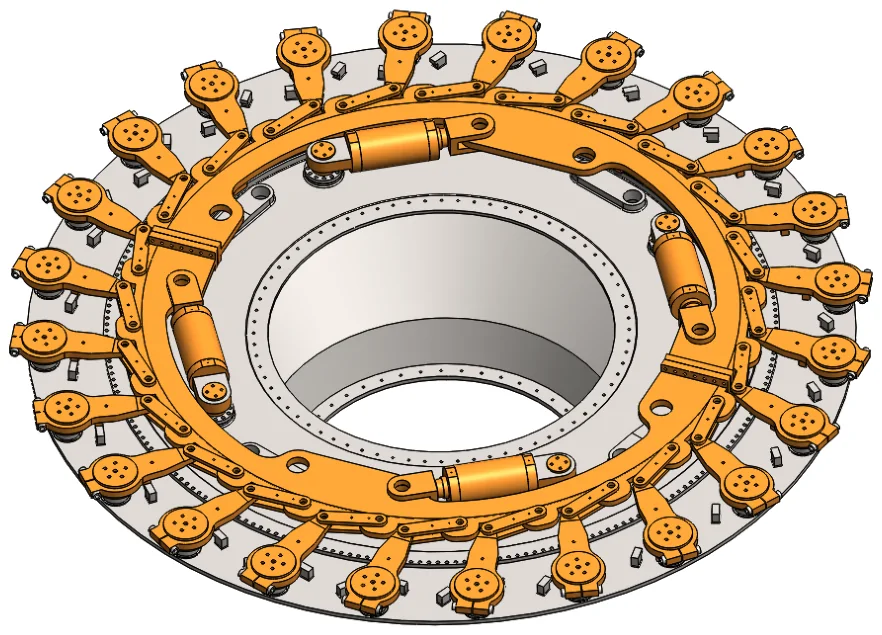
Three-dimensional model of the regulating ring assembly developed.

The geometric model of the regulating ring assembly was developed based on original engineering drawings, assembly schematics, technical documentation, and field measurements performed at Sinop Energia. The objective was to obtain a geometrically representative model capable of reproducing the global structural behavior of the regulating system under operational loading conditions. Figure 2 presents the resulting three-dimensional model of the regulating ring assembly.

The model includes the regulating ring, connecting arms, support structures, actuator interfaces, and other components directly responsible for transmitting mechanical loads throughout the distributor mechanism. Particular attention was given to the interfaces between structural components, since these regions govern the overall stiffness and load distribution within the assembly.

In order to reduce computational complexity, components not directly associated with the global structural response of the regulating ring were simplified or omitted. Internal mechanisms, small fastening elements, lubrication systems, and secondary mechanical details were not explicitly represented. A fully detailed representation of all mechanical subsystems would considerably increase the geometric complexity of the model, resulting in a substantially larger finite element mesh and higher computational costs. Since the objective of this study is to evaluate the overall stress distribution of the regulating ring rather than the local behavior of individual components, these simplifications were considered appropriate and do not significantly affect the global structural response of interest.

The resulting three-dimensional model provides a faithful representation of the actual assembly while maintaining a level of complexity compatible with large-scale finite element simulations. The model serves as the basis for the subsequent structural analyses and sensor placement studies developed in this work.

### 2.3. Planimetric and Altimetric Survey

A planimetric and altimetric survey was conducted to characterize the current geometric condition of the regulating ring and establish reference measurements for the displacement monitoring system. The survey was performed along the entire circumference of the regulating ring, considering the 24 distributor sectors responsible for controlling the water flow supplied to the Kaplan turbine.

**Figure 3:**
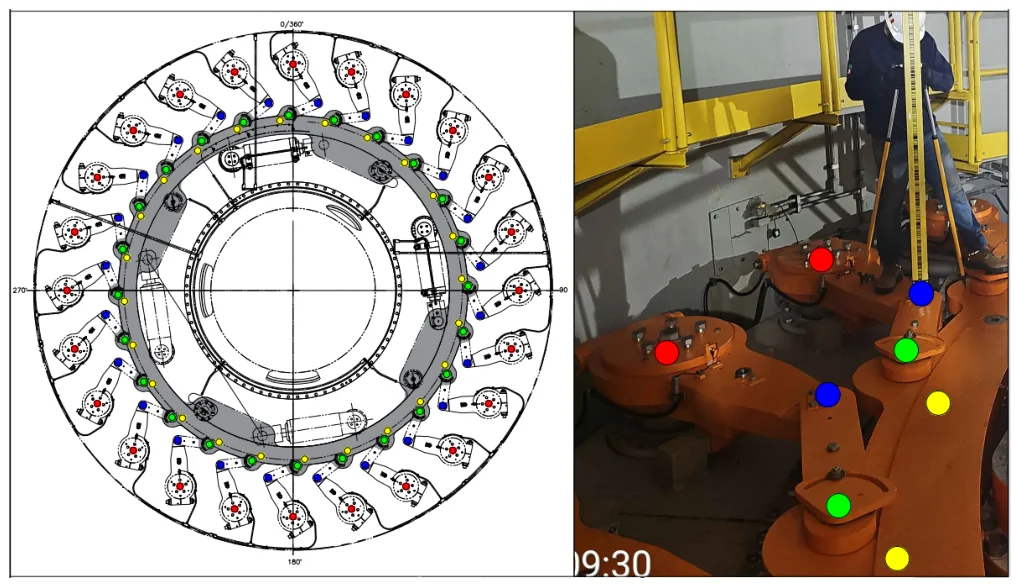
Measurement locations used in the planimetric and altimetric survey. The highlighted regions correspond to the ring surface (yellow), guide vane position adjustment point (green), upper connecting rod (blue), and lever cover (red).

As illustrated in Figure 3, four measurement locations were defined within each distributor sector: the ring surface, guide vane position adjustment point, upper connecting rod, and lever cover. These locations were selected to provide a representative description of the geometric configuration of the regulating mechanism while allowing the relative displacements between adjacent components to be quantified.

Measurements were acquired under two operating conditions corresponding to the extreme positions of the regulating ring: 0% opening, representing the fully closed distributor condition, and 100% opening, representing the fully open condition. Evaluating both positions allowed the complete operating range of the regulating mechanism to be characterized and provided valuable information regarding the displacement patterns experienced during operation.

Beyond assessing the current condition of the regulating ring assembly, the survey results were also employed to define the reference positions for the proximity sensors used in the monitoring system. These measurements establish the baseline geometry of the structure, enabling future sensor readings to be interpreted as deviations from the measured reference condition. Furthermore, the collected data served as an important input for the finite element model calibration and for the subsequent development of the proposed framework.

### 2.4. Finite Element Model of the Regulating Ring

The three-dimensional model developed in the previous stage was used as the basis for the finite element analyses. The objective of this stage was to create a numerical model capable of reproducing the structural behavior of the regulating ring under real operating conditions, while maintaining compatibility with the subsequent sensor placement and framework development procedures.

#### 2.4.1. Material Properties

The regulating ring assembly is composed of structural components manufactured from ASTM A516 Grade 70. The mechanical properties adopted in the simulations are summarized in Table 1. The material properties were obtained directly from the corresponding standards and manufacturer specifications [22].

Regarding welded regions, the mechanical properties of the weld material were assumed to be equal to or greater than those of the base material. Therefore, the welds were not modeled as distinct material regions, and the corresponding structural components were considered homogeneous throughout the numerical analyses.

#### 2.4.2. Finite Element Model and Boundary Conditions

**Figure 4:**
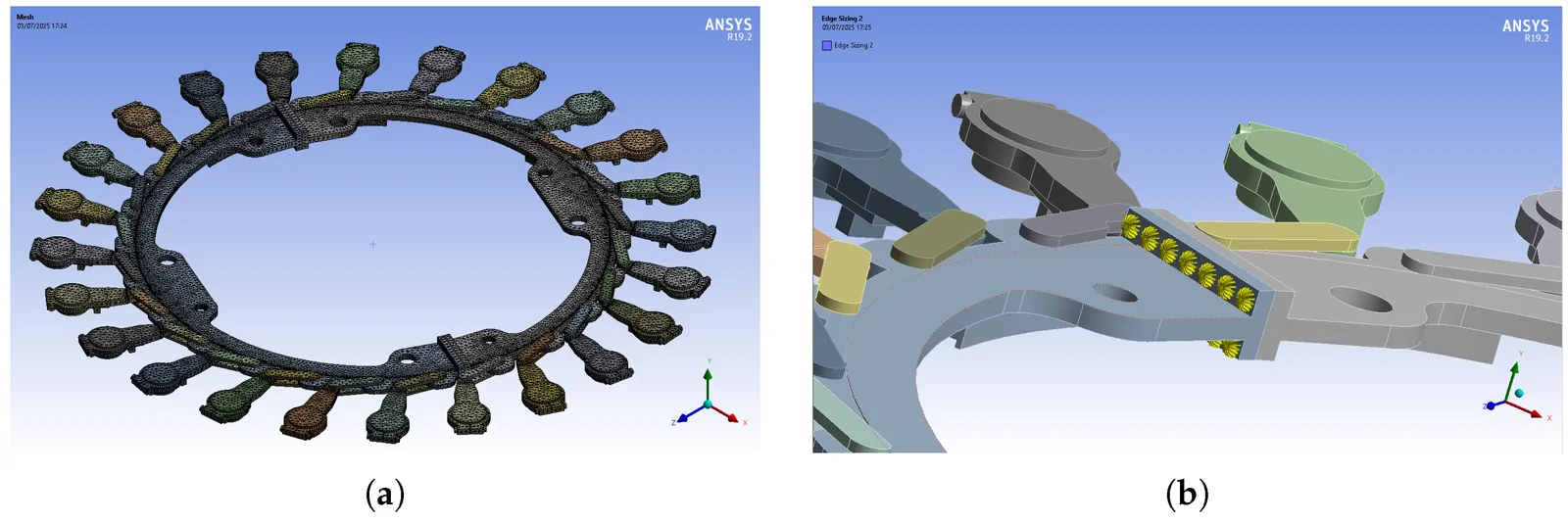
Finite element mesh adopted for the structural analyses: (**a**) global mesh of the regulating ring assembly; (**b**) locally refined mesh in a critical structural region.

The finite element model was generated directly from the three-dimensional geometry presented in Section 2.2. All numerical simulations were performed using ANSYS Mechanical 19.0. Initially, a global finite element mesh was created to discretize the regulating ring assembly, as shown in Figure 4a. To improve the accuracy of the structural analyses, local mesh refinement was applied in regions expected to exhibit higher stress gradients, including geometric discontinuities, structural interfaces, and load transfer regions. A detailed view of one of these refined regions is presented in Figure 4b. This strategy allows increased solution accuracy in critical locations while maintaining a computational cost compatible with large-scale parametric analyses.

**Figure 5:**
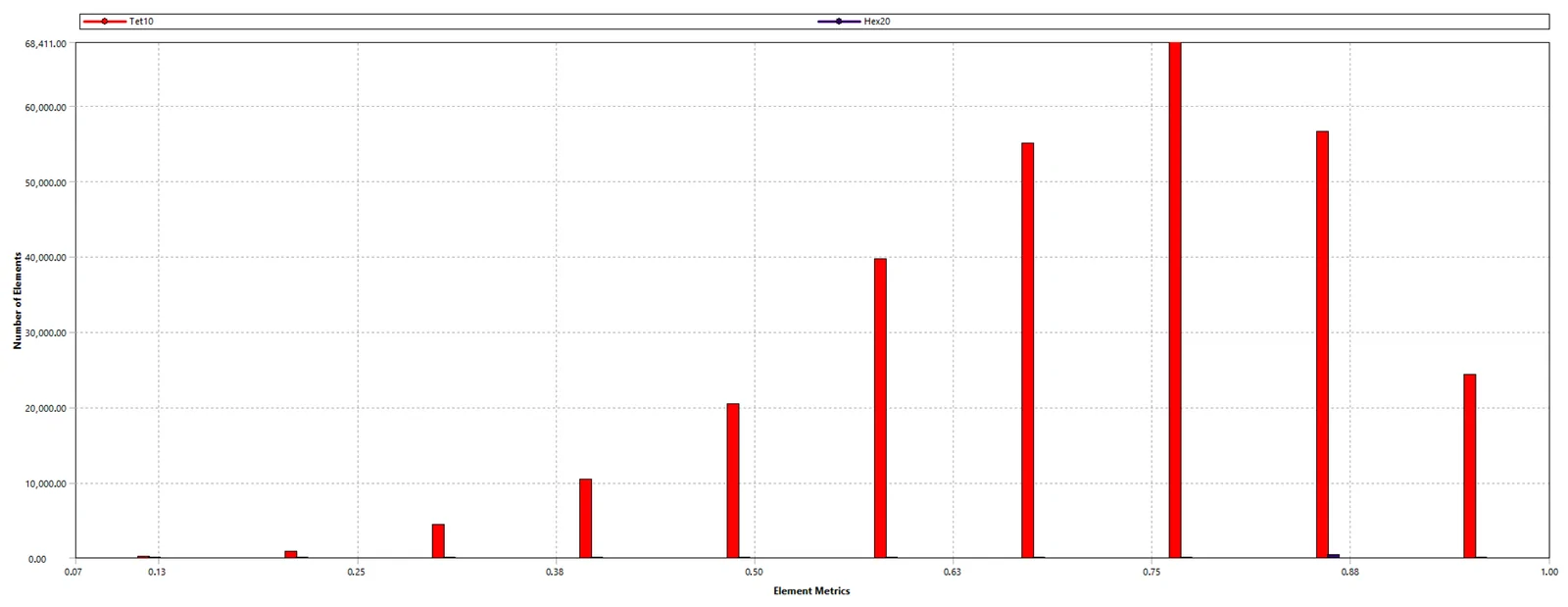
Mesh quality assessment of the finite element model.

The quality of the generated mesh was subsequently evaluated using the standard mesh quality metrics available in the finite element software. As illustrated in Figure 5, the adopted mesh presents quality indicators within the recommended range, ensuring adequate element distortion and numerical stability throughout the simulations. Therefore, the generated mesh was considered suitable for all subsequent structural analyses.

The bolted connections were represented using beam elements combined with bolt pretension loads. This approach preserves the stiffness contribution and preload effects of the bolts while avoiding the substantial increase in computational cost associated with detailed three-dimensional bolt geometries.

Loads generated by the hydraulic servomotors were applied at the corresponding connection points of the regulating ring. The magnitude of these loads was obtained from field measurements acquired through the instrumentation installed on the hydraulic cylinders, ensuring that the numerical model accurately reproduced the actual operating conditions of the hydroelectric unit.

Displacement boundary conditions were imposed through Remote Displacement constraints applied to the regulating ring and lever arms. The prescribed displacement values were obtained directly from the planimetric and altimetric survey described in Section 2.3, allowing the measured geometric condition of the structure to be incorporated into the finite element model. Consequently, the numerical analyses represent the actual configuration of the regulating mechanism rather than an idealized geometry.

Surface interactions between the regulating ring and the Orkot bearing material, as well as between the two ring halves, were modeled using frictional contacts with a friction coefficient of 0.6. The articulated connections between the ring, connecting rods, and levers were represented through revolute joints, allowing relative rotational motion and reproducing the kinematic behavior of the regulating mechanism. Finally, the support region of the assembly was modeled using fixed support boundary conditions, restricting all translational and rotational degrees of freedom.

To evaluate the structural response under the extreme operating conditions of the distributor mechanism, two initial simulation scenarios were considered. The first corresponds to the fully closed distributor condition (0% opening), while the second represents the fully open distributor condition (100% opening). These analyses provided an initial assessment of the stress distribution and deformation patterns throughout the regulating ring and served as the basis for the subsequent sensor placement study.

### 2.5. Proximity Sensor Selection and Installation

The proposed monitoring strategy is based on inductive proximity sensors installed to continuously measure the vertical displacement of the regulating ring during operation. Compared with conventional strain gauges or displacement transducers, inductive proximity sensors provide a simple, robust, and cost-effective solution for long-term monitoring in industrial environments. Their non-contact operating principle minimizes mechanical wear, while their high resistance to vibration and harsh environmental conditions makes them particularly suitable for hydroelectric power plants.

The selected sensor was the IFM ZX1 inductive displacement sensor (ifm electronic gmbh, Essen, Germany), which operates over a measurement range of 0–15 mm with an analog output of 0–10 V. According to the manufacturer specifications, the sensor provides an accuracy of ±0.1% of the measurement range, repeatability better than 0.05 mm, response time below 1 ms, IP67 protection rating, and stainless-steel housing, ensuring reliable operation under the environmental conditions encountered in hydroelectric generating units.

**Figure 6:**
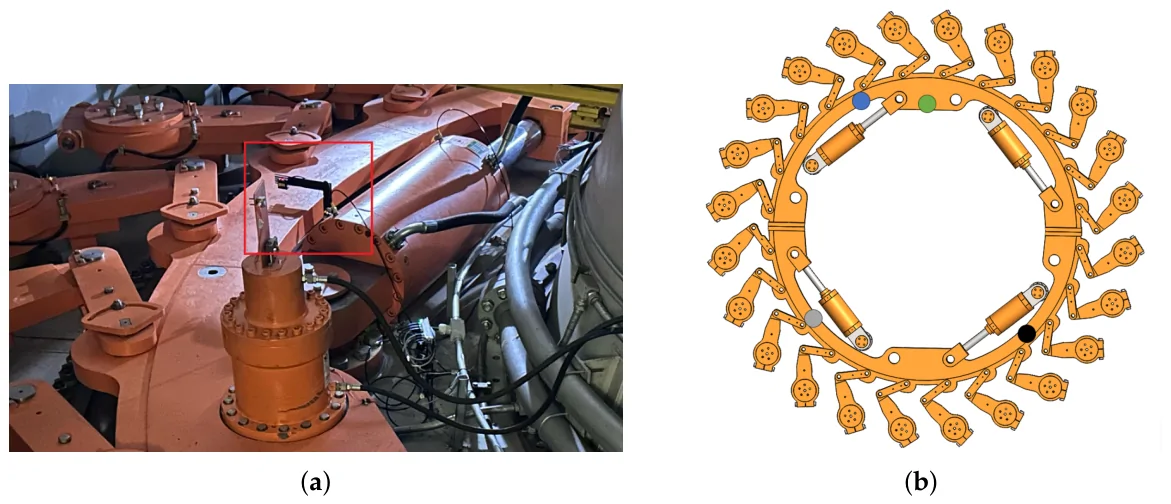
Installation and positioning of the inductive proximity sensor used to monitor the vertical displacement of the regulating ring: (**a**) inductive proximity sensor installed in the regulating ring assembly (The red box indicates the region where the sensor is installed); (**b**) sensor positioning and installation location.

The sensors were installed to measure the vertical displacement of the regulating ring, as illustrated in Figure 6. This measurement direction was selected because vertical displacements are directly associated with the structural deformation of the ring under operational loading, providing valuable information regarding its mechanical condition.

A total of four proximity sensors were installed at distributor sectors 6, 8, 18, and 23. The selection of these monitoring locations was based on the operational experience and engineering knowledge of the technical staff at the Sinop Energia.

To ensure consistency between the experimental measurements and the numerical model, the reference positions established during the planimetric and altimetric survey described in Section 2.3 were adopted as the baseline for all proximity sensor measurements. Consequently, the displacement readings obtained during operation are referenced to the same geometric datum used in the finite element model, allowing direct comparison between measured and simulated structural responses.

The use of proximity sensors offers additional advantages for continuous structural monitoring. Besides their relatively low acquisition and installation costs, these sensors require minimal maintenance, can be easily integrated into existing supervisory systems, and enable real-time acquisition of displacement data without interfering with the operation of the generating unit. These characteristics make them particularly attractive for online structural monitoring applications, where continuous and reliable data acquisition is essential.

### 2.6. Machine Learning Model Development

This section presents the methodology adopted to develop the machine learning surrogate model used in the proposed framework. The objective is to replace the computationally expensive finite element analyses with a data-driven model capable of estimating the complete stress distribution of the regulating ring from a limited number of proximity sensor measurements.

The proposed methodology comprises four main stages. First, a finite element database is generated using a Design of Experiments (DOE) approach to efficiently sample the operating conditions of the regulating ring. Subsequently, the generated stress fields are preprocessed and standardized to produce a consistent dataset suitable for machine learning. Next, Principal Component Analysis is employed to reduce the dimensionality of the stress field representations while preserving their dominant spatial characteristics. Finally, a single Random Forest regression model is trained on the pooled dataset to establish the relationship between the guide vane opening, the measured sensor displacements, and the reduced-order representation of the stress fields, enabling the reconstruction of the complete von Mises stress distribution in near real time.

### 2.7. Representative Operating Conditions Adopted to Generate the Finite Element Simulation Database

The finite element model developed in the previous sections provides an accurate representation of the structural behavior of the regulating ring. However, each high-fidelity simulation requires a relatively long computational time, making direct finite element analyses unsuitable for real-time structural monitoring during the operation of the hydroelectric generating unit.

To overcome this limitation, the proposed framework adopts a surrogate modeling strategy. Instead of solving the finite element model online, a machine learning model is trained offline using a database generated from finite element simulations. Once trained, the surrogate model is capable of estimating the complete stress distribution of the regulating ring from a limited number of proximity sensor measurements in only a few seconds, enabling online monitoring while preserving the predictive capability of the numerical model.

The quality of the surrogate model strongly depends on the representativeness of the training database. Although increasing the number of finite element simulations generally improves model generalization, each additional simulation introduces a considerable computational cost. Consequently, an efficient strategy is required to generate a representative set of operating conditions while maintaining the number of simulations within practical limits.

Considering the four displacement sensors adopted in this study and three representative displacement levels assigned to each sensor (4, 7, and 10 mm), a full-factorial sampling would require $3^{4}=81$ finite element simulations for each guide vane opening. Since eleven operating positions, ranging from 0% to 100% in increments of 10%, were considered, a complete factorial design would result in a total of 891 finite element simulations. Such computational effort was considered impractical for the present application.

Therefore, a structured representative sampling strategy, inspired by the principles of Design of Experiments (DOE), was adopted to generate the training database [23,24,25]. Rather than exhaustively evaluating every possible combination of sensor displacements, four representative operating conditions were selected for each guide vane opening. The selected combinations were distributed across the low, medium, and high displacement levels of all four sensors to provide representative coverage of the expected operating domain while substantially reducing the computational burden. The operating conditions were selected based on engineering judgment, considering the expected structural behavior of the regulating mechanism and the objective of adequately spanning the range of practical operating conditions.

The resulting sampling plan is presented in Table 2. In total, only 44 finite element simulations were required, representing a reduction of approximately 95% compared with a complete factorial design. Considering that each high-fidelity finite element simulation requires several hours of computational time, performing all 891 simulations would demand an impractically large computational effort. The adopted representative sampling strategy therefore provided an effective compromise between computational cost and coverage of the operating domain, generating a representative training database while keeping the total simulation time within practical limits.

For all finite element simulations, the stress contour images were exported using a fixed color scale. The minimum and maximum stress values of the colormap were kept constant throughout the entire dataset, ensuring that each RGB color corresponded to the same von Mises stress level in every simulation. This standardization is essential for the subsequent image-based machine learning procedure, as it guarantees that identical colors represent identical physical quantities across all samples. Without this normalization, variations in the automatically adjusted color scale could introduce inconsistencies in the training data, leading the learning algorithm to associate the same color with different stress values. The unified color scale adopted, ranging from 0 to 4100 MPa, which establishes the correspondence between each RGB color and its associated von Mises stress value.

#### 2.7.1. Data Preprocessing

The finite element simulations generated through the DOE procedure were used to construct a surrogate model capable of estimating the complete stress distribution of the regulating ring directly from proximity sensor measurements. Instead of executing a computationally expensive finite element simulation for each new operating condition, the proposed framework learns the relationship between the measured displacements and the corresponding stress field through supervised machine learning. Once trained, the model can estimate the stress distribution in a few seconds, making the methodology suitable for online structural monitoring applications.

The proposed framework requires two types of input information: (i) the current guide vane opening and (ii) the displacement measurements acquired by the four inductive proximity sensors installed at distributor sectors 6, 8, 18, and 23. Rather than training a separate surrogate for each discrete opening condition, the guide vane opening was incorporated directly as an additional input variable of a single, unified regression model. This design choice allows the surrogate to interpolate continuously across the full operating range of the distributor mechanism, avoiding the need to select among multiple discrete models and enabling the model to exploit shared structural behavior across neighboring opening conditions during training.

A pixel-wise machine learning model such as the one adopted in this work is only meaningful if a given pixel location consistently represents the same physical region of the regulating ring across all simulations. However, the raw stress contour images exported directly from the finite element post-processor are not guaranteed to be geometrically aligned, since the position and apparent scale of the ring within the rendered viewport can vary slightly between simulations. To address this limitation, a dedicated geometric normalization procedure was applied to every image prior to model training. First, the component of interest was automatically segmented from the white background of the rendering by thresholding the average pixel intensity, followed by binary morphological opening and closing operations to suppress residual noise and thin shadow artifacts. The bounding box of the segmented region was then used to crop the image tightly around the ring, which was subsequently rescaled, preserving its original aspect ratio, and centered on a fixed 512×512 white canvas with a 5% margin. This alignment step ensures that a given pixel coordinate corresponds to the same physical location of the regulating ring in every sample of the dataset, which is an essential prerequisite for the pixel-wise dimensionality reduction performed by PCA, as described in the following subsection. As noted in Section 2, the color scale used to render the stress contours was likewise held fixed throughout the dataset, so that image alignment and color standardization jointly guarantee that identical pixel values encode identical physical quantities across all simulations.

After the alignment procedure, each 512×512×3 RGB image was converted into a one-dimensional vector of 786,432 elements to enable the application of the dimensionality reduction algorithm described in the following subsection.

#### 2.7.2. Principal Component Analysis

Principal Component Analysis is one of the most widely used statistical techniques for dimensionality reduction and feature extraction [26]. The method transforms a set of correlated variables into a new orthogonal coordinate system whose axes, called principal components, are ordered according to the amount of variance explained by the original data. Consequently, most of the information contained in a high-dimensional dataset can often be represented using only a relatively small number of principal components.

In engineering applications, PCA has been extensively employed in structural health monitoring, image processing, and surrogate modeling because it allows high-dimensional datasets to be represented in a compact latent space while preserving their dominant spatial characteristics [26,27]. In the present work, PCA is employed to reduce the dimensionality of the finite element stress fields, enabling the regression model to estimate only a limited number of coefficients instead of the complete stress image.

(1) $$Y=\left[\begin{array}{c} y_{1}\\ y_{2}\\ \vdots \\ y_{N} \end{array}\right],$$

where *N* denotes the total number of finite element simulations and each vector $y_{i}$ contains the pixel values associated with one stress field.

PCA projects the original dataset onto a lower-dimensional latent space according to

(2) Z=YW,

where W is the matrix containing the principal components and Z contains the corresponding PCA scores.

Only the retained principal components were used as output variables during model training. This strategy substantially reduces the dimensionality of the regression problem while preserving the dominant features of the stress distributions. Besides reducing computational cost, PCA also removes redundancy among neighboring pixels, improving the robustness and generalization capability of the subsequent machine learning model.

A single PCA model was fitted using the complete set of stress field images obtained from all guide vane openings, rather than fitting an independent PCA for each operating condition. The number of retained components was not fixed a priori; instead, it was automatically determined for each fold by requiring the cumulative explained variance to reach at least 95%. This variance-based criterion ensures that the latent representation retains the dominant spatial patterns of the stress fields while adapting the dimensionality of the latent space to the actual complexity of the training data.

#### 2.7.3. Random Forest Regression

The reduced PCA coefficients were estimated using a Random Forest regressor. Random Forest is an ensemble learning algorithm originally proposed by Breiman [28], consisting of a collection of decision trees trained using bootstrap aggregation (bagging) and random feature selection. Each decision tree is independently trained from a random subset of the available training samples and input variables, while the final prediction is obtained by averaging the outputs of all trees.

Compared with a single decision tree, Random Forest presents improved generalization capability, greater robustness against noisy measurements, and reduced susceptibility to overfitting [28,29]. Furthermore, RF can accurately model highly nonlinear relationships without requiring assumptions regarding the statistical distribution of the data, making it particularly attractive for engineering regression problems involving complex structural responses.

Rather than constructing an independent model for each discrete guide vane opening, a single Random Forest model was trained on the pooled dataset covering the entire operating range of the regulating mechanism, from 0% to 100% opening. In this formulation, the guide vane opening is treated as an additional predictor alongside the four sensor displacements, allowing the model to learn how the stress distribution evolves continuously with the opening condition rather than being restricted to a discrete set of pre-defined operating points. This strategy avoids the need for a model-selection step during online operation and enables the surrogate to leverage structural similarities between neighboring operating conditions during training.

The Random Forest learns the nonlinear mapping

(3) $$f:\left[a,s_{1},s_{2},s_{3},s_{4}\right]\to \left[z_{1},z_{2},\ldots ,z_{k}\right],$$

where *a* denotes the guide vane opening, $s_{1}$ to $s_{4}$ are the displacements measured by the four proximity sensors installed at distributor sectors 6, 8, 18, and 23, and $z_{i}$ represents the PCA coefficients associated with the corresponding stress field.

The models were implemented using the Scikit-learn machine learning library [30]. To select the number of decision trees, a sensitivity analysis was performed by training the Random Forest with {5,10,25,50,100,250,500} estimators and evaluating the reconstruction accuracy (MSE, RMSE, PSNR, and SSIM) obtained through a four-fold cross-validation scheme, in which, for each guide vane opening, three of the four representative operating conditions of Table 2 were used for training and the remaining one for testing. The reconstruction accuracy improved rapidly with the number of trees and stabilized beyond 100 estimators, with negligible gains obtained from further increasing the ensemble size. Based on this analysis, 100 decision trees were adopted for the final model, providing a favorable trade-off between predictive accuracy and computational cost, while the remaining hyperparameters were maintained at their default Scikit-learn values.

The generalization capability of the trained surrogate to entirely unseen operating conditions was subsequently assessed through a leave-one-opening-out cross-validation procedure. In each round, all images corresponding to one guide vane opening were completely withheld from training, while the PCA and Random Forest models were fitted using the remaining ten openings; the withheld opening was then used exclusively for testing. This procedure was repeated for all eleven opening conditions (0% to 100%, in increments of 10%), providing a stringent evaluation of the model’s ability to interpolate the stress field for operating conditions not observed during training.

After validation, a final Random Forest model, together with a corresponding global PCA model, was retrained using the complete dataset (all 44 finite element simulations, covering the 11 guide vane openings and the 4 representative sensor combinations per opening), so as to make full use of the available finite element simulations for deployment in the monitoring platform. For this final model, the PCA representation retained 10 components, corresponding to a cumulative explained variance of 96.93%, consistent with the 8–9 components obtained across the individual leave-one-opening-out folds. Fitting the global PCA required approximately 5.0 s, while training the final Random Forest model required approximately 0.12 s, both negligible compared with the several hours required by a single high-fidelity FEM simulation.

The Random Forest predicts the PCA coefficients associated with the input operating condition. These coefficients are subsequently used to reconstruct the complete stress field through the inverse PCA transformation,

(4) $$\hat{Y}=\hat{Z}W^{T}+\bar{Y},$$

where $\hat{Z}$ denotes the vector of PCA coefficients estimated by the Random Forest model, W is the matrix containing the retained principal components, and $\bar{Y}$ is the mean image computed during the PCA fitting process.

The inverse transformation reconstructs the original high-dimensional representation of the stress field as a one-dimensional vector containing the RGB values of all image pixels. This vector is subsequently reshaped into its original dimensions of 512×512×3, recovering the complete color image representing the predicted von Mises stress distribution.

Because all finite element simulations were generated using the same fixed stress color scale, each RGB value corresponds to a unique von Mises stress level throughout the entire dataset. Consequently, the reconstructed image preserves both the spatial distribution of the stresses and their quantitative magnitude, allowing direct comparison with the finite element simulations without additional normalization or color rescaling.

Finally, the reconstructed stress image is displayed by the monitoring platform, providing an intuitive visualization of the predicted structural condition of the regulating ring in near real time.

## 3. Results and Discussion

The performance of the proposed framework is evaluated through a comprehensive analysis of its prediction accuracy, computational efficiency, and practical applicability. The sensitivity of the reconstruction accuracy to the size of the Random Forest ensemble is first examined to justify the number of decision trees adopted in the final model. The reconstructed stress fields are then compared with the corresponding finite element solutions using quantitative image similarity metrics obtained through a leave-one-opening-out cross-validation procedure. The dimensionality reduction achieved through Principal Component Analysis is subsequently analyzed to assess the compactness of the learned representation, followed by an evaluation of the relative contribution of the guide vane opening and of each proximity sensor to the prediction process. The final model, trained on the complete dataset, is then presented, and its reconstruction capability is illustrated qualitatively for one representative operating condition. Finally, the engineering implications, advantages, and limitations of the proposed framework are discussed, highlighting its potential for real-time structural health monitoring of hydroelectric generating units.

### 3.1. Random Forest Sensitivity Analysis

To determine the number of decision trees adopted in the final Random Forest model, a sensitivity analysis was performed using the four-fold cross-validation procedure described in Section 2. For each guide vane opening, the four representative operating conditions listed in Table 2 were partitioned into four folds, such that three samples were used for training and the remaining sample was used for testing. This procedure was repeated until each sample had served once as the test set. Consequently, the reported performance for each Random Forest configuration corresponds to the average reconstruction accuracy over all four folds and all eleven guide vane openings.

The number of decision trees was varied over {5,10,25,50,100,250,500}, while all remaining Random Forest hyperparameters were kept unchanged. For each configuration, the reconstructed stress fields were compared with the corresponding FEM solutions using the Mean Squared Error, Root Mean Squared Error, Peak Signal-to-Noise Ratio, and Structural Similarity Index Measure. The resulting average metrics are summarized in Table 3.

The results indicate that the Random Forest reconstruction performance is only weakly sensitive to the number of decision trees over the investigated range. Across all cross-validation folds, the reconstruction accuracy varies only slightly, with MSE values between approximately 177 and 190, PSNR between 27.4 and 28.2 dB, and SSIM between 0.938 and 0.940. The absence of a clear monotonic trend indicates that the ensemble reaches a stable prediction regime with a relatively small number of trees, after which additional estimators provide only marginal improvements.

Although the configuration with 500 decision trees achieved the lowest average MSE and the highest SSIM, the improvement over the 100-tree model is negligible when averaged across the four cross-validation folds and the eleven guide vane openings. Increasing the ensemble size beyond 100 trees therefore leads primarily to higher computational cost, with little practical benefit in reconstruction accuracy.

### 3.2. Leave-One-Opening-Out Sensitivity Analysis

The reconstruction capability of the proposed framework was evaluated through a leave-one-opening-out cross-validation procedure, in which each guide vane opening (0% to 100%, in increments of 10%) was entirely withheld from training and used exclusively as a test set, as described in Section 2. This procedure provides a stringent assessment of the ability of the single global surrogate model to reconstruct stress fields for operating conditions that were never observed during training, rather than restricting the evaluation to a single representative example.

The MSE, RMSE, PSNR, and SSIM were computed for the test images of each withheld opening. Table 4 summarizes the resulting metrics per opening condition, together with the number of retained PCA components and the corresponding cumulative explained variance. Across all eleven withheld openings, the surrogate model achieved an average PSNR of 17.4 dB and an average SSIM of 0.837, with the PCA representation requiring 8 to 9 components to reach over 95% of the cumulative explained variance.

Compared with the accuracy previously obtained when independent PCA and Random Forest models were fitted separately for each opening, the leave-one-opening-out results are more conservative, since the model is now required to generalize to an operating condition it has never observed, rather than interpolating within data from the same opening. Nonetheless, the reconstructed stress fields preserve the overall spatial distribution of the FEM solutions, and the achieved computational cost (approximately 1.5 s per stress field, against several hours for a full FEM simulation) confirms that the single global surrogate remains compatible with near real-time online operation.

### 3.3. PCA Compression

The dimensionality reduction achieved by the proposed framework was evaluated by analyzing the number of principal components required to reach a cumulative explained variance of at least 95%, considering the single global PCA model fitted with all stress field images. Since each stress field originally consists of hundreds of thousands of pixels, the PCA representation aims to preserve the dominant spatial information while drastically reducing the number of variables required by the machine learning model.

Across the eleven folds of the leave-one-opening-out cross-validation, the number of retained components ranged from 8 to 9, with an average of 8.9 components and an average cumulative explained variance of 96.6% (Table 4). These results indicate that the stress fields exhibit a highly redundant spatial structure, allowing the vast majority of the relevant information to be represented by fewer than ten principal components, even when the PCA model must generalize to an entirely unseen operating condition.

This dimensionality reduction is one of the key factors enabling the computational efficiency of the proposed framework. By reconstructing the complete stress fields from fewer than ten predicted principal component coefficients per prediction, instead of the full pixel-wise representation, the methodology achieves an average inference time of approximately 1.5 s per stress field, making near real-time structural monitoring feasible. Consequently, the Random Forest regressor estimates only the dominant mechanical modes instead of attempting to predict hundreds of thousands of individual pixel values, substantially improving learning efficiency and reducing the risk of overfitting.

### 3.4. Sensor Importance

An additional advantage of the proposed Random Forest-based framework is the possibility of evaluating the relative contribution of each input variable to the prediction of the PCA coefficients. Since the Random Forest algorithm provides a feature importance measure based on the reduction of prediction error achieved by each input variable during tree construction, the importance of the guide vane opening and of each of the four proximity sensors was extracted from the final global model. The obtained results are presented in Table 5.

The results reveal a markedly unbalanced distribution of importance among the five input variables. The guide vane opening alone accounts for 96.9% of the total importance, whereas the four proximity sensors jointly contribute only 3.1%, with sensor S2 (1.7%) being the most informative of the four and sensor S1 (0.4%) the least. This behavior is a direct consequence of the relative variability of each input variable within the training database: the guide vane opening spans the entire 0–100% operating range and is therefore the dominant driver of the overall stress magnitude and spatial distribution, whereas the sensor displacements are confined, by design of the DOE described in Section 2, to a comparatively narrow range of three representative levels (4, 7, and 10 mm) intended to capture second-order variations around each opening rather than the primary loading condition.

This finding should not be interpreted as an indication that the proximity sensors are dispensable. Rather, it clarifies the physical role played by each input: the guide vane opening establishes the coarse, dominant operating regime of the regulating ring, while the sensor displacements provide the fine-grained, condition-specific corrections that differentiate the stress field among the four representative combinations sampled at a given opening. Because the impurity-based importance used by Random Forest tends to favor variables with larger explanatory range, the reported values should be interpreted as a measure of relative predictive leverage under the sampled operating envelope rather than as a definitive statement on the mechanical relevance of each sensor. A complementary analysis based on permutation importance, or on an expanded DOE with a wider range of sensor displacements for each opening, would help disentangle the coarse and fine-grained contributions more rigorously and is left for future work.

From a practical standpoint, this result nonetheless carries a relevant implication for the framework’s architecture: since the guide vane opening is typically available from the plant’s supervisory control and data acquisition (SCADA) system with high reliability, the surrogate model remains informative even under partial sensor degradation, while the proximity sensors remain necessary to capture the local structural response associated with asymmetric loading of the regulating ring that the opening alone cannot resolve.

### 3.5. Direct Sensor Perturbation Analysis

To complement the impurity-based feature importance reported above, a direct perturbation analysis was performed to evaluate how the reconstructed stress field responds to controlled variations of each individual proximity sensor. For every guide vane opening (0% to 100%, in increments of 10%), a reference image was generated with all four sensors fixed at 7 mm, the central value of the sampled range. Each sensor was then varied independently from 1 to 10 mm, in steps of 1 mm, while the remaining three sensors were kept at the 7 mm reference value. For every perturbed configuration, the reconstructed image was compared with the reference image of the corresponding opening using the MSE and SSIM, providing a direct, model-agnostic measure of sensor sensitivity, independent of the internal splitting criterion of the Random Forest.

**Figure 7:**
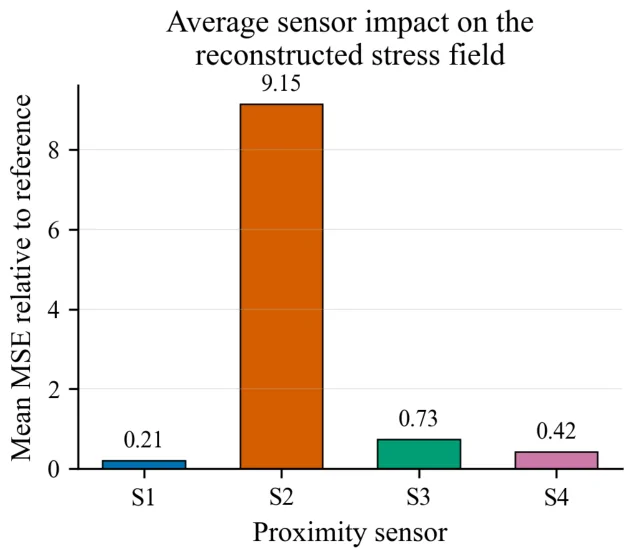
Average sensor impact on the reconstructed stress field.

**Figure 8:**
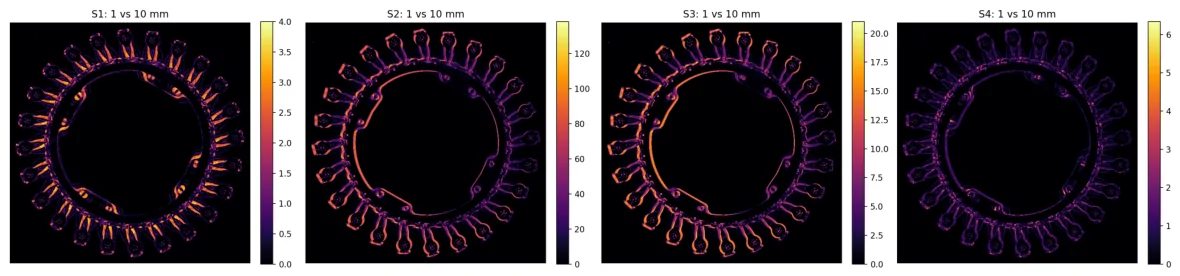
Spatial distribution of the perturbation effect of each sensor (opening = 50%).

Table 6 summarizes the resulting metrics, averaged over the eleven guide vane openings and the full 1–10 mm perturbation range of each sensor. Figure 7 presents the corresponding average MSE per sensor, and Figure 8 illustrates the spatial distribution of the perturbation effect for the representative 50% opening condition.

The perturbation analysis is consistent with the Random Forest feature importance reported above: sensor S2 produces the largest average change in the reconstructed image (mean MSE of 9.15, mean SSIM of 0.997), followed by S3 (0.73) and S4 (0.42), with S1 exerting the smallest influence (0.21). The agreement between this direct, model-agnostic perturbation approach and the internal impurity-based ranking reinforces the conclusion that the four proximity sensors contribute unequally to the reconstructed stress field, with S2 consistently identified as the most informative of the four. Interestingly, this outcome is also consistent with the operational experience of the plant’s technical staff, who have long regarded the sector monitored by S2 as one of the most mechanically active regions of the regulating ring, lending additional practical support to the sensitivity ranking obtained from the surrogate model.

The spatial difference maps in Figure 8 further indicate that this sensitivity is not spatially uniform: perturbing S2 and S3 produces more pronounced changes along the ring body and the base of the connecting arms, while S1 and S4 mainly affect the arm tips, with comparatively smaller magnitude. This pattern is broadly consistent with the physical installation of the sensors at distributor sectors 6, 8, 18, and 23, suggesting that each sensor is preferentially informative about the structural response of the regions closer to its own installation point.

It should be noted that, owing to the discrete sensor levels used in the Design of Experiments (4, 7, and 10 mm, Section 2), the response of the surrogate model to intermediate sensor values is not perfectly smooth, occasionally showing localized, non-monotonic variations rather than a gradual trend. This behavior is consistent with the sparse and comparatively narrow sampling of the sensor displacements already discussed as a limitation of the present dataset, and further supports the recommendation to expand the sensor-displacement sampling in future iterations of the framework, so as to improve the smoothness and reliability of the surrogate’s response across the full input domain.

### 3.6. Final Model Trained on the Complete Dataset

The results presented in the previous subsections were obtained through cross-validation procedures, in which one guide vane opening (Section 3) or one sensor combination (Random Forest sensitivity analysis) was withheld at a time for testing purposes. Once the number of decision trees (100) and the overall modeling approach were validated in this manner, the final PCA–Random Forest surrogate model was retrained using the complete dataset, comprising all 44 stress field images generated for the eleven guide vane openings and their representative sensor combinations.

When fitted with the complete dataset, the PCA model retained 10 principal components, corresponding to a cumulative explained variance of 96.93%, consistent with the range of 8 to 9 components observed across the individual cross-validation folds. The Random Forest regressor was likewise trained on the full set of PCA coefficients using 100 estimators, as established in Section 3. This final model, rather than any of the individual cross-validation folds, constitutes the surrogate model recommended for deployment in the proposed framework’s architecture, since it leverages the entire available database to estimate stress fields for any combination of guide vane opening and sensor readings within the sampled operating envelope.

### 3.7. Image Reconstruction Performance

**Figure 9:**
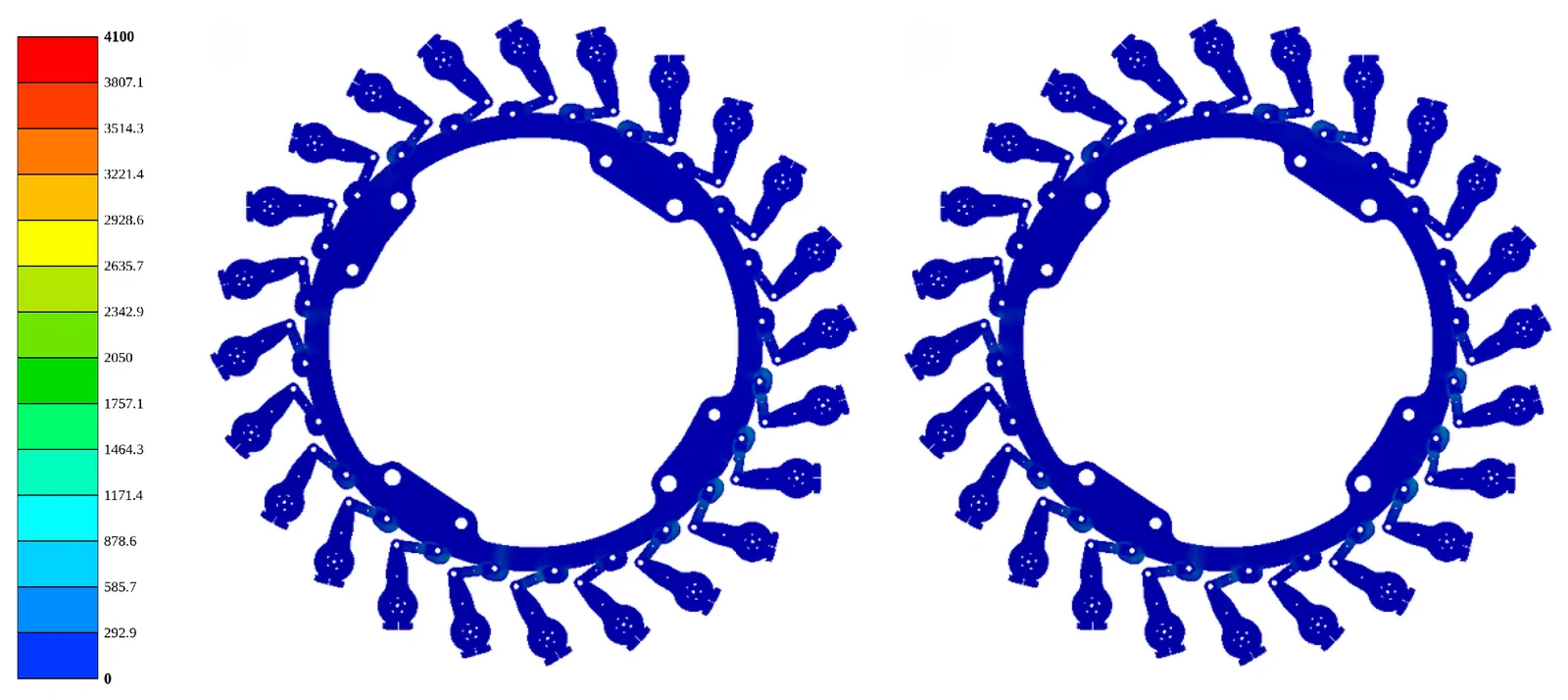
Comparison between the original FEM stress field (**left**) and the corresponding reconstructed image generated by the proposed framework (**right**) for one representative operating condition randomly selected from the dataset.

To qualitatively illustrate the reconstruction capability of the final model trained on the complete dataset, Figure 9 presents one representative operating condition randomly selected from the generated database, comparing the FEM-generated and the reconstructed stress fields for the upper surface of the regulating ring. Both contour images share the unified color scale described in Section 2, which maps each RGB color to a von Mises stress value in the 0–4100 MPa range and thus allows the two fields to be compared directly and interpreted quantitatively.

The reconstructed stress field preserves the overall spatial distribution and magnitude of the FEM solution, illustrating the qualitative agreement achieved by the surrogate model. Combined with the quantitative results reported in Section 3 and an inference time of approximately 1.5 s per stress field, this example confirms that the final model is compatible with near real-time online operation.

### 3.8. Engineering Implications and Practical Considerations

The results obtained throughout this study demonstrate that the proposed framework extends beyond an image reconstruction methodology, constituting a practical structural monitoring tool for hydropower generating units. Unlike conventional finite element analyses, which require high computational effort and are therefore restricted to offline engineering studies, the proposed surrogate model enables continuous estimation of the complete stress field directly from a limited number of displacement measurements acquired during plant operation.

From an engineering perspective, one of the most relevant findings is that the structural behavior of the regulating ring can be inferred from the guide vane opening together with only four proximity sensors, without requiring dense instrumentation of the structure. This result indicates that the deformation patterns of the regulating mechanism exhibit strong spatial correlations, allowing the machine learning model to recover unmeasured stress regions from a compact set of inputs. Such capability is particularly attractive for industrial applications, where installation costs, accessibility, wiring complexity, and maintenance requirements often limit the number of available sensors.

The proposed methodology also illustrates the complementary roles of physics-based and data-driven modeling. Rather than replacing the finite element model, machine learning acts as an efficient surrogate trained from high-fidelity numerical simulations. Consequently, the predictive capability of the finite element model is preserved while reducing the computational cost by several orders of magnitude. Considering that a conventional three-dimensional FEM simulation typically requires several hours to complete, whereas the proposed framework reconstructs the stress field in approximately 1.5 s, the methodology becomes compatible with continuous online monitoring and real-time decision support.

Another important observation concerns the low intrinsic dimensionality of the stress fields. Despite each stress map containing hundreds of thousands of RGB values, the PCA analysis demonstrated that fewer than ten principal components (8 to 9, depending on the fold) are sufficient to preserve approximately 96.6% of the relevant structural information. This behavior suggests that the deformation of the regulating ring is governed by a limited number of dominant mechanical modes, which considerably simplifies the regression problem while improving computational efficiency and model robustness.

The feature importance analysis further provides valuable engineering insight into the selected monitoring configuration. Rather than a balanced contribution among all inputs, the results showed that the guide vane opening dominates the prediction of the overall stress level, while the four sensors jointly refine the estimate to capture the specific loading condition among the representative combinations sampled at each opening. This finding supports a layered interpretation of the monitoring architecture, in which SCADA-derived operating parameters and dedicated structural sensors play complementary, rather than equivalent, roles. Moreover, the proposed methodology could support future sensor placement optimization studies, for instance by evaluating permutation importance or by expanding the DOE with a wider range of sensor displacements per opening, so as to more clearly isolate the structural information carried by each monitoring location.

Despite the encouraging results, some limitations should be acknowledged. The surrogate model was trained using a finite number of numerical simulations generated through the adopted Design of Experiments strategy. Consequently, prediction accuracy outside the sampled operating domain cannot be guaranteed. Furthermore, the present study considers a single generating unit equipped with four displacement sensors and focuses exclusively on the reconstruction of von Mises stress fields. Because the sensor displacements were sampled over a comparatively narrow range (4, 7, and 10 mm) relative to the full 0–100% span of the guide vane opening, the resulting feature importance analysis is dominated by the opening variable; a wider or denser sampling of sensor displacements would be required to more precisely quantify the individual contribution of each proximity sensor.

Overall, the proposed framework represents an effective compromise between computational efficiency and predictive capability. By combining high-fidelity finite element simulations with machine learning-based surrogate modeling, the methodology provides sufficiently accurate stress field estimation to support predictive maintenance, early fault detection, and operational decision-making, contributing to increased reliability and availability of hydroelectric generating units.

## 4. Conclusions

This work presented an FEM-based machine learning framework for online estimation of the stress field of a hydropower regulating ring by integrating high-fidelity finite element simulations with machine learning techniques. The proposed methodology combines a single global Principal Component Analysis model for dimensionality reduction with a single Random Forest regression model, trained on the guide vane opening and four proximity sensor measurements, to reconstruct the complete von Mises stress distribution across the entire operating range of the distributor mechanism.

The obtained results, validated through a leave-one-opening-out cross-validation procedure, demonstrated that the reconstructed stress fields preserve the overall spatial distribution of the FEM solutions even when an entire guide vane opening is withheld from training, achieving an average PSNR of 17.4 dB and SSIM of 0.837 across the eleven tested openings. The PCA analysis further revealed that the structural response of the regulating ring can be represented using fewer than ten principal components (8 to 9, accounting for approximately 96.6% of the cumulative explained variance), substantially reducing the complexity of the learning problem while preserving the dominant deformation patterns. A sensitivity analysis of the Random Forest ensemble size showed that 100 decision trees achieve accuracy comparable to considerably larger ensembles, supporting the adopted configuration as a favorable trade-off between accuracy and computational cost.

The feature importance analysis of the final model indicated that the guide vane opening is by far the dominant predictor, reflecting the narrower range over which the sensor displacements were sampled relative to the full operating range of the opening. This ranking was independently corroborated by a direct, model-agnostic perturbation analysis, in which each sensor was varied individually while the others were held fixed: the same sensor identified as most informative by the Random Forest importance measure also produced the largest change in the reconstructed stress field, and the associated spatial difference maps indicated that its influence is concentrated near its own installation sector on the ring. This outcome is consistent with the operational experience of the plant’s technical staff, providing additional practical support for the sensitivity ranking obtained from the surrogate model and reinforcing the physical plausibility of the learned relationship between sensor displacements and the reconstructed stress field.

From a practical standpoint, the proposed framework enables near real-time structural assessment by reducing the computational time from several hours required by conventional finite element analyses to approximately 1.5 s. This computational efficiency makes the methodology suitable for continuous structural monitoring, predictive maintenance, and operational decision support in hydroelectric generating units, while the leave-one-opening-out results provide a realistic estimate of the surrogate’s ability to generalize to previously unobserved operating conditions.

Future work will focus on expanding the simulation database with a wider range of sensor displacements per guide vane opening to better isolate the individual contribution of each sensor and to improve the smoothness of the surrogate’s response across the full input domain, incorporating operational measurements acquired during long-term plant operation, investigating adaptive model updating strategies to narrow the accuracy gap observed under the leave-one-opening-out validation, and evaluating the proposed framework across a broader set of operating conditions and sensor configurations. These developments will further enhance the robustness and generalization capability of the proposed framework for industrial structural health monitoring applications.

## Figures and Tables

**Table 1:** Mechanical properties adopted in the finite element model [22].

Yield Strength (MPa)	Ultimate Strength (MPa)	Young’s Modulus (GPa)	Density (kg/m^3^)	Poisson’s Ratio (−)
260	485	210	7859	0.30

**Table 2:** Design of Experiments adopted to generate the finite element simulation database for training the machine learning model.

Opening (%)	Sensor 1 (mm)	Sensor 2 (mm)	Sensor 3 (mm)	Sensor 4 (mm)
0	4	4	4	4
0	7	7	10	4
0	10	10	7	4
0	10	10	7	7
10	4	7	10	4
10	7	10	4	7
10	10	4	7	10
10	4	10	7	7
20	7	4	10	7
20	10	7	4	10
20	4	10	7	4
20	7	7	10	10
30	10	4	4	7
30	4	7	10	10
30	7	10	7	4
30	10	7	10	4
40	4	10	4	10
40	7	4	7	4
40	10	7	10	7
40	4	7	7	10
50	4	7	7	7
50	7	10	4	7
50	10	4	10	7
50	7	7	4	10
60	10	10	4	4
60	4	7	7	10
60	7	4	10	7
60	10	7	4	10
70	4	4	10	7
70	7	7	4	10
70	10	10	7	4
70	4	10	10	7
80	4	10	10	10
80	7	4	7	10
80	10	7	4	10
80	4	4	10	7
90	7	10	7	4
90	10	4	10	7
90	4	7	4	10
90	7	4	10	10
100	10	7	7	10
100	4	10	10	4
100	7	4	4	7
100	10	10	7	4

**Table 3:** Sensitivity of the reconstruction accuracy to the number of decision trees in the Random Forest model.

Number of Trees	MSE	RMSE	PSNR (dB)	SSIM
5	180.87	11.49	28.22	0.9400
10	184.94	11.83	27.67	0.9384
25	190.02	12.07	27.41	0.9380
50	187.74	11.98	27.46	0.9386
100	179.91	11.64	27.76	0.9400
250	179.94	11.64	27.71	0.9394
500	177.11	11.51	27.83	0.9401

**Table 4:** Leave-one-opening-out cross-validation results for the global PCA–Random Forest surrogate model (average over the withheld test images of each opening).

Opening (%)	Components	Variance Explained	MSE	RMSE	PSNR (dB)	SSIM
0	9	0.9655	1088.9	33.00	17.76	0.848
10	9	0.9684	1033.0	32.14	17.99	0.853
20	9	0.9662	1129.5	33.61	17.60	0.839
30	9	0.9663	1175.2	34.28	17.43	0.840
40	9	0.9642	1238.2	35.19	17.20	0.830
50	8	0.9660	1599.8	39.35	16.36	0.809
60	9	0.9645	1139.9	33.61	17.64	0.818
70	9	0.9669	1217.6	34.89	17.28	0.833
80	9	0.9661	1191.0	34.51	17.37	0.845
90	9	0.9691	1233.0	35.11	17.22	0.844
100	9	0.9659	1244.4	35.27	17.18	0.843
Average	8.9	0.9663	1208.2	34.63	17.37	0.837

**Table 5:** Feature importance obtained from the final global Random Forest model.

Input Variable	Importance
Opening (*a*)	0.9685
S1	0.0039
S2	0.0172
S3	0.0057
S4	0.0047

**Table 6:** Average impact of each proximity sensor on the reconstructed stress field, obtained by perturbing each sensor over its 1–10 mm range while holding the remaining sensors at the 7 mm reference value (averaged over all eleven guide vane openings).

Sensor	Mean MSE	Max MSE	Mean SSIM	Min SSIM
S1	0.21	1.19	0.9998	0.9993
S2	9.15	459.87	0.9972	0.8889
S3	0.73	12.18	0.9996	0.9974
S4	0.42	3.54	0.9996	0.9987

## Data Availability

The data presented in this study are available on request from the corresponding author.
